# Socio-economic resources, young child feeding practices, consumption of highly processed snacks and sugar-sweetened beverages: a population-based survey in rural northwestern Nicaragua

**DOI:** 10.1186/s12889-015-1374-5

**Published:** 2015-01-21

**Authors:** Mariela Contreras, Elmer Zelaya Blandón, Lars-Åke Persson, Anders Hjern, Eva-Charlotte Ekström

**Affiliations:** Department of Women’s and Children’s Health, Uppsala University, SE-751 85 Uppsala, Sweden; Asociación para el Desarrollo Económico y Social de El Espino (APRODESE), Chinandega, Nicaragua; Centre for Health Equity Studies, Karolinska Institutet/Stockholm University, Stockholm, Sweden

**Keywords:** Education, Food security, Socioeconomic, Young child feeding

## Abstract

**Background:**

Socio-economic resources may be associated with infant feeding in complex patterns in societies undergoing a nutrition transition. This study evaluates associations of housing quality, food security and maternal education to the World Health Organization (WHO) feeding recommendations and to consumption of highly processed snacks (HP snacks) and sugar-sweetened beverages (SSBs) in rural Nicaragua.

**Methods:**

Data were collected from May to November 2009, with mothers of 0- to 35-month-olds being asked about young child feeding using a food frequency questionnaire. A validated questionnaire was used to assess household food insecurity and data were collected on maternal education and housing quality. Pearson’s chi-squared test was used to compare proportions and determine associations between the resources and young child feeding. The three socio-economic resources and other confounders were introduced to multivariate logistic regression analyses to assess the independent contribution of the resources to the feeding practices and consumption of HP snacks and SSBs.

**Results:**

Mothers with the lowest education level were more likely to be exclusively breastfeeding (EBF) their infants (OR not EBF: 0.19; 95% CI: 0.07, 0.51), whilst mothers of 6- to 35-month-olds in the lowest education category had more inadequate dietary diversity (DD) (OR for not meet DD: 2.04; 95% CI: 1.36, 3.08), were less likely to consume HP snacks (OR for HP snacks: 0.47; 95% CI: 0.32, 0.68) and SSBs (OR for SSBs: 0.68; 95% CI: 0.46, 0.98), compared to mothers with the highest level of education. Similarly, children residing in households with the highest food insecurity were also more prone to have inadequate dietary diversity (OR for not meet DD: 1.47; 95% CI: 1.05, 2.05). The odds for double burden of suboptimal feeding (concurrent inadequate diet and consumption of HP snacks/SSBs) were significantly lower in children of least educated mothers (OR: 0.64; 95% CI: 0.44, 0.92).

**Conclusions:**

Higher level of education was associated with both more and less adherence to the WHO recommended feeding practices as well as with more consumption of HP snacks and SSBs. Regardless of educational strata, the children in the community were exposed to suboptimal feeding practices conducive to both under- as well as overnutrition.

## Background

Child undernutrition continues to be a significant influence in global health that, together with zinc and vitamin A deficiency and inappropriate breastfeeding habits, contributes to 45% of all child deaths in low- and middle-income countries [1,2]. The World Health Organization (WHO) has issued guidelines aimed at the improvement of infant and young child feeding practices to ultimately lead to better child nutrition worldwide [3,4]. Whilst undernutrition remains a global health problem, the trend is that the numbers of overweight and obese children are increasing [5], are often concurrent in the same population [6] and occur even in the same individuals who are affected by undernutrition [7]. Although the WHO infant and young child feeding guidelines promote appropriate feeding behaviours that should be practised during the first years of life to prevent undernutrition, these recommendations do not fully address the nutrition transition dilemma with its characteristic increase in the consumption of high-energy-dense food combined with a sedentary lifestyle. Early consumption of highly processed snacks (HP snacks) and sugar-sweetened beverages (SSBs) are usually high in total energy, saturated fats and sugars or salt and may contribute to children being overweight [8,9] and their consumption has emerged even in societies undergoing the early stages of the nutrition transition.

A considerable body of research has addressed underlying resources as being of key importance for the recommended child feeding behaviours. Knowledge, skills and other traits, such as intentions and attitudes that are acquired and adopted along with education, have been studied in relation to recommended infant and young child feeing practices [10,11]. However, knowledge and skills may not be sufficient to guarantee the implementation of good infant and young child feeding practices. Beyond the exclusive breastfeeding period, mothers must also have access to quality foods to feed their children. Therefore, household food security as an indicator of food accessibility has been associated with, for example, dietary adequacy [12]. The household wealth or assets, as indicators of the household economy, have also been independently associated with infant and young child feeding [13,14].

In research focusing on low- and middle-income countries, considerably less attention has been paid to how resources are associated with the consumption of foods and beverages that generally are considered unhealthy, such as HP snacks and SSBs, and results have so far been partly contradictory. Higher household asset scores in Botswana were associated with higher levels of adolescent unhealthy eating behaviours, suggesting that better economic conditions do not necessarily translate into better nutrition. Nor do lower socio-economic resources translate into better feeding practices. In Brazil, 48-month-old children of poorer households with mothers of lower education level consumed more sweets, chocolates and crispy salty snacks [15]. Results so far indicate that the associations between the consumption of HP snacks and SSBs and socio-economic resources are context- and age-specific and thus may depend on the current stage of the nutrition transition.

Determinants of recommended feeding behaviours might not be the same as determinants of not recommended or non-favourable feeding behaviours. Analyses of both perspectives are needed to inform policy and practice. Household wealth, household food security and level of maternal education are indicators of socio-economic resources that are often associated with each other. However, as they may capture different pathways and thus display different entry points for nutrition-sensitive interventions, it is important to evaluate their independent associations with both recommended and not recommended feeding behaviours.

Nicaragua is a lower-middle-income [16] Central American country. Whilst few studies addressing infant and young child feeding in Nicaragua have been published, it has been documented that frequency of exclusive breastfeeding is low and childhood stunting is relatively common [17]. The country is currently undergoing a nutrition transition [18] with its characteristic changes in food habits and it is likely that an understanding of socio-economic resources that contribute to both recommended and not recommended feeding behaviours are of importance for infant and young child nutrition. In this study, we aimed to determine the independent associations between household wealth, household food insecurity and level of maternal education as determinants of recommended feeding behaviours as well as of feeding of HP snacks and SSBs amongst infants and children aged 0–35 months in rural northwestern Nicaragua. We hypothesised that higher levels of the socio-economic resources would be associated with better dietary diversity, but also with non-favourable feeding behaviours such as low exclusive breastfeeding and higher consumption of HP snacks and SSBs amongst the children.

## Methods

### Study setting and population

This cross-sectional study was conducted in the municipalities of Santo Tomás del Nance, San Juan de Cinco Pinos, San Pedro del Norte and San Francisco del Norte, Department of Chinandega in rural northwestern Nicaragua with a population of around 25,000 people and about 5,000 households. The area is collectively called *Los Cuatro Santos* (The Four Saints), where most of the population is engaged in small-scale subsistence farming activities whilst other income-generating opportunities are limited. Most of the mothers in the area were occupied with household activities and childcare and did not have a stable source of income. The Asociación para el Desarrollo Económico y Social de El Espino (APRODESE) is a local non-governmental economic and social development organization that has developed projects in *Los Cuatro Santos* to improve living standards. To inform the *Los Cuatro Santos* society about recent progress in the reduction of child mortality, all households in the area also received a pamphlet containing general health messages, as well as recommendations on infant feeding. In 2003, a Health and Demographic Surveillance System (HDSS) was established by APRODESE to monitor the effects of the development projects. Three rounds of HDSS data collection were performed in 2003, 2007 and 2009 respectively. Data for the current study were collected from May to November 2009 and included child feeding and household food insecurity data in addition to the routinely collected socio-demographic information.

### Study sample and data collection

The study sample consisted of children aged 0–35 months (0–3 years). It is common for infant and young child feeding studies to focus on children younger than 2 years, given that efforts to promote better nutrition may have more impact during the first 1,000 days of a child’s life [2]. However, we also included children aged 24–35 months because they may also be at risk of undernutrition [19] as was reconfirmed in a recent study [20]. Further, this age category may be more prone to be exposed to HP snacks and SSBs, underlining the relevance to evaluate non-favourable feeding behaviours in such a group.

**Figure 1:**
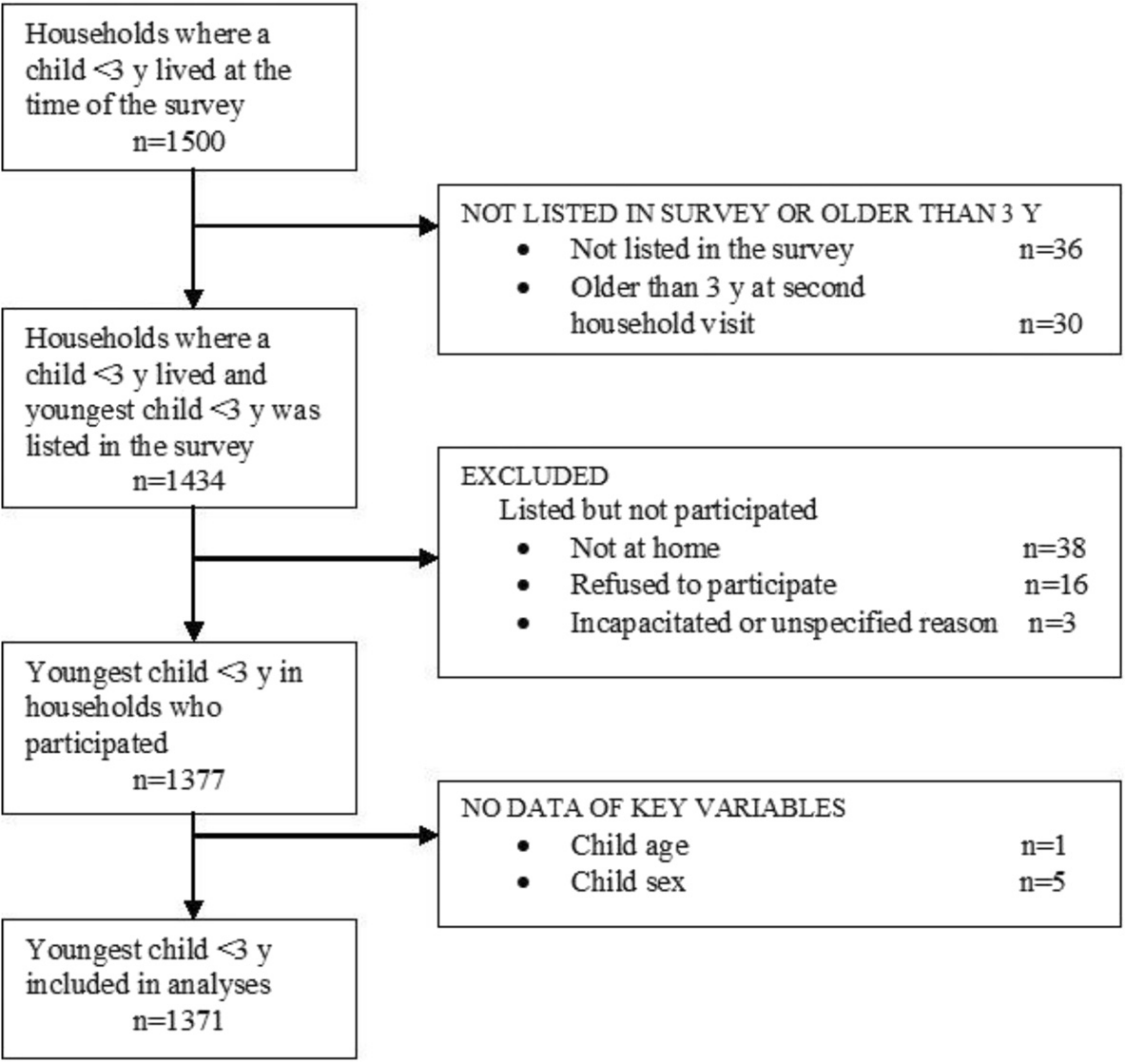
**Participation flow of child feeding and nutrition study in Nicaragua.** Children in households who did not participate were those not listed in the first household visit and those who were older than 3 years in the second household visit. Other children in households did not participate because they were not found at home, refused to participate, were incapacitated or gave no specific reasoning for not participating. A few children in households had missing information on key variables and were excluded.

The organization of data collection started with a group of locally recruited and trained interviewers who visited all households (about 5,000) in the area and collected socio-demographic and household food insecurity data. They identified and listed households with at least one child younger than 3 years. Thereafter, a second group of interviewers revisited the 1,500 households fulfilling this criterion and collected information on infant and young child feeding practices and the consumption of HP snacks and SSBs of the youngest child in the household (Figure 1).

### Infant and young child feeding

The instruments to assess recommended infant and young child feeding practices were developed in accordance with the WHO core feeding indicators using a food frequency questionnaire (FFQ), recalling the number of times a food or drink item was consumed during the last 24 hours [3]. In all, 70 food items and 11 beverages were organized into seven groups, as suggested in the WHO guidelines [3,4], and were included in the FFQ list. The FFQ was pilot-tested in a nearby community [21] before being used in the survey. The food and beverage items, cookies and crackers, chocolates and candies, salty crisps, carbonated soft drinks, sweetened powdered fruit drinks and coffee, were listed in the FFQ to represent items being high-energy-dense and sugary or salty. To assess meal frequency, a specific question was asked regarding the number of eating episodes per day for the child in accordance with the, at that time, available WHO guidelines [3].

The WHO guidelines were used to construct feeding indicators on exclusive breastfeeding for children aged 0–5 months and on continued breastfeeding for children aged 12–15 months [3]. Complementary feeding indicators intended for children aged 6–23 months (but here also used for the 24- to 35-month-olds) were based on the following seven food groups: (a) grains, roots, tubers; (b) legumes and nuts; (c) vitamin-A fruits and vegetables; (d) other fruits and vegetables; (e) meats; (f) eggs; and (g) dairy products [3]. Fruits and vegetables were categorised and grouped according to their vitamin A content using food composition tables [22]. One point was given to a food group when at least one of its food items was consumed the previous day. The points for the food groups were summed to give a score that ranged from 0–7. A cut-off of four food groups or more was defined as achieving minimum dietary diversity [3]. Further, the minimum meal frequency indicator was created and included breastfed children who had eaten the minimum number of times or more (2 times if aged 6–8 months, and 3 times if aged 9–35 months) and non-breastfed children who ate or were given milk feeds the minimum number of times or more (4 times for children aged 6–35 months) the previous day [3]. The WHO guidelines were also used to construct an overall dietary adequacy indicator. Thus, for breastfed infants, the minimum acceptable diet included those who had at least the minimum dietary diversity and minimum meal frequency. For non-breastfed children to reach minimum dietary adequacy they should, in addition to these criteria, have consumed at least two milk feedings [3].

Further, one HP snack and one SSB variable were developed from counting whether any of the HP snacks and SSBs were consumed at least once, followed by the creation and categorisation into two dichotomous HP snack and SSB variables. Sugary coffee, however, was not included in the SSB variable, as it is a common drink item and deeply rooted in the culture and, as such, may have different determinants than the other items.

Children aged 6–35 months with at least one inadequate complementary feeding practice and concurrent consumption of any HP snack or SSB were defined as being exposed to a “double burden of suboptimal feeding”.

### Household economic resources

Housing quality was used as a proxy for the household’s overall economic resources. The basic needs approach (i.e. Index of Unsatisfied Basic Needs) has been commonly used in Latin American settings [23] and is partly based on the presence and type of drinking water source, house floor and walls, electricity supply and the presence of a latrine or toilet. Given that we sought to give the same value of importance to each resource level, the pre-coded options were all coded from 1 to 3, where a value of 1 meant the lowest housing quality situation and 3 the highest. For electricity supply, however, the variable was dichotomous (yes/no) and 1 point was given to households who had no electricity, whilst a value of 3 was given to households with electricity. The pre-coded options were summed up to create a housing quality score ranging from 5–15, which was further divided into tertiles; lowest (5–10), middle (11–12) and highest (13–15).

### Maternal education

Maternal education was assessed by asking the highest level of schooling completed by the mothers at the time of the survey. The questions were based on a previous survey, with 28 potential pre-coded options included in the questionnaire. These were defined by the non-governmental organization (NGO) APRODESE and were part of the HDSS data collection instrument. The level of education was further divided into three categories. To reflect the Millenium Development Goal 2 of at least 5 years of primary education for all, the lowest level of education was defined as having less than 5 years of schooling. The middle level of education was having 5 to 9 years of schooling, whilst the highest was 10 years or more.

### Household food security

Household food insecurity was measured using the Household Food Insecurity Access Scale (HFIAS), which has been validated in various international settings [24]. The scale showed good internal consistency in our sample with Cronbach’s α = .863 [25]. This instrument consists of nine questions that measure uncertainty around obtaining food, limited access to high-quality foods and reduction of food quantity in the past 4 weeks [24]. The pre-coded options were *never* (0 points), *rarely* (once or twice in the past 4 weeks; 1 point), *sometimes* (3 to 10 times in the past 4 weeks; 2 points) and *often* (more than 10 times in the past 4 weeks; 3 points). The score of the answers to the questions was summed up (0–27) and thus a higher value signified a worse condition with more household food insecurity [24]. For descriptive purposes of occurrence of food insecurity, the categorisation of food insecurity followed the HFIAS guide and was defined as food secure, mildly food insecure, moderately food insecure and severely food insecure, based on a number of assumptions that were made by the HFIAS authors, given there is no universally accepted approach to defining the cut-offs [24]. For the inferential analyses, we created a continuous variable of food insecurity which was categorised into tertiles of the score defined as lowest (0–7), middle (8–11) and highest (12–27) food insecurity.

### Maternal occupation

Maternal occupation was assessed by an open-ended question that asked the type of occupation for people older than 5 years in the household. The most common occupations of mothers were housewife, teacher, office worker and health worker. Other maternal occupations, but less common, were agriculturist, artisan and student. For the inferential analyses, we divided maternal occupation into two categories: the first being unemployed (e.g. housewife) mothers and the second labelled as employed (e.g. teacher, health worker) mothers.

### Statistical analysis

Prevalence of the feeding practices and consumption of HP snacks and SSBs were described across the three levels of the three underlying resources in the focus of this study. Cross-tabulation with the Pearson’s chi-squared test was used to compare proportions and evaluate the association between maternal education and maternal occupation. Cross-tabulations were also used to compare proportions and determine whether resources were associated with the recommended feeding practices, consumption of HP snacks and/or SSBs and as well as a double burden of suboptimal feeding. Three statistical models were tested to evaluate associations after adjustment for potential confounders and to further understand the independent associations with the resources. The first model included the unadjusted results, the second included only the potential confounders whilst the third model included both the potential confounders and all three resources of interest in order to evaluate their independent associations with the feeding practices, consumption of HP snacks and SSBs and a double burden of suboptimal feeding. Factors associated with both exposure and outcome, which changed the effect estimate by 10% or more were included in the feeding practices, consumption of HP snacks and SSBs and double burden of suboptimal feeding models. These factors were maternal age, child age and municipality. The resources and confounders were introduced to the multivariate logistic regression analyses, which then provided unadjusted and adjusted odds ratios calculated with 95% confidence intervals for the feeding practices, consumption of HP snacks and SSBs and double burden of suboptimal feeding. The variables in the multivariate logistic regression were coded so that an odds ratio below one should be interpreted as a favourable outcome, whilst an odds ratio above one should be regarded as unfavourable. Significance level was defined at <0.05 and all statistical analyses were completed using the Statistical Package for the Social Sciences version 20 [26].

### Ethics

Our study followed the protocol of the Universal Helsinki Declaration. The Biomedical Research Ethics Committee at the University of Nicaragua in León approved the study protocol. Informed consent was obtained from all participating mothers and caretakers.

## Results

Ninety-two percent of the eligible households with at least one child aged younger than 3 years were available for data collection (Figure 1). The main reasons for omissions were that the children had not yet been listed at the first round of household visits or that they were not found at home despite up to three repeated visits. Further, children who had passed the age of eligibility at the subsequent visit were excluded from statistical analyses. A few questionnaires with missing information on key variables were also excluded. Of a total of 1,377 households and children originally included, 1,371 were included in the final analyses.

### General characteristics

A low proportion of the households in *Los Cuatro Santos* had access to tap water (22.2%), whilst a relatively high proportion had latrines or toilets (75.7%) (Table 1). In spite of an agricultural tradition in the area, few households had a home garden in use (8.0%). Food insecurity was high, with more than one-third of the households reporting to be severely food insecure and more than one-half moderately so. More than two-thirds of the mothers had between 5 to 9 years of schooling and one-fifth had 10 years or more. Most of the mothers were housewives (89.5%) and were either married or lived with a partner (87.2%). Less than one percent of the mothers worked in agricultural activities (data not shown).

**Table 1:** **General household, maternal and child characteristics in Nicaragua**

**Characteristics (n = 1,371)**	**%** ^**1**^	**n/N** ^**1,2**^
**Household**		
Housing quality		
Tap water	22.2	305/1371
Latrine/toilet	75.7	1037/1371
Soil/Earth floor	68.1	933/1371
Wall of adobe/brick/wood	98.0	1343/1371
Electricity	78.5	1076/1371
Home garden in use	8.0	108/1352
Food insecurity		
Severely food insecure	36.0	494/1371
Moderately food insecure	51.0	700/1371
Mildly food insecure	6.9	95/1371
Food secure	6.0	82/1371
**Maternal**		
Age category (year)		
<20	12.8	175/1366
20-29	48.6	664/1366
30-39	30.7	420/1366
40+	7.8	107/1366
Education		
<5 years	35.8	488/1364
5-9 years	43.1	588/1364
≥10 years	21.1	288/1364
Marital status		
Single/divorced/widow	12.8	176/1371
Married/with partner	87.2	1195/1371
Occupation		
Teacher/health worker/office worker	5.4	73/1364
Other (i.e. student, artisan)	5.1	70/1364
Housewife	89.5	1221/1364
**Child**		
Age category (month)		
0-5	16.8	231/1371
6-11	18.2	250/1371
12-23	32.2	441/1371
24-35	32.7	449/1371
Gender		
Girls	50.8	697/1371

^1^Values are percentages (%) and (n/N); ^2^Variation in denominator is due to missing data.

### Recommended feeding practices

#### Breastfeeding in infants aged 0–5 months and children aged 12–15 months

Indices of infant and young child feeding practices disaggregated by socio-economic resources are shown in Table 2. Whilst exclusive breastfeeding amongst infants 0- to 5-months-old was low, it was significantly more common amongst infants of mothers with a lower level of education (Table 2). After adjustment for potential confounders, as well as for the other socio-economic resources (Table 3), the odds for exclusive breastfeeding remained highest in infants of mothers with the lowest education. No independent associations were observed between exclusive breastfeeding and neither household food insecurity nor housing quality, making maternal education the most important resource for exclusive breastfeeding.

**Table 2:** **Prevalence of recommended and not recommended feeding practices, by socio-economic resources in Nicaragua**

			**WHO feeding indicators** ^**1**^					
	**Exclusive ** **breast feeding** **0–5 m**	**Continued ** **breast feeding** **12–15 m**	**Minimum dietary diversity** ^**2**^ **6-35 m**	**Minimum meal frequency** ^**3**^ **6-35 m**	**Minimum acceptable diet** **6–35 m**	**Highly processed snacks** **6–35 m**	**Sugar-sweetened ** **beverages** **6–35 m**	**Double burden of suboptimal feeding** ^**4**^ **6-35 m**
**Socio-economic resources**	**% (n/N)**	**% (n/N)**	**% (n/N)**	**% (n/N)**	**% (n/N)**	**% (n/N)**	**% (n/N)**	**% (n/N)**
All	34 (209)	78 (162)	67 (1135)	61 (1125)	40 (1125)	59 (1135)	36 (1135)	38 (1135)
Housing quality^5^								
Lowest	39 (30/78)	81 (47/58)	62 (274/445)	61 (267/437)	37 (163/437)	54 (242/445)	32 (143/445)	38 (168/445)
Middle	37 (28/75)	74 (46/62)	69 (298/431)	61 (263/430)	41 (177/430)	62 (265/431)	36 (155/431)	38 (164/431)
Highest	24 (14/58)	79 (33/42)	71 (188/264)	58 (153/262)	44 (114/262)	61 (162/264)	41 (108/264)	37 (98/264)
	*p* = 0.17	*p* = 0.66	*p* = 0.012	*p* = 0.73	*p* = 0.24	*p* = 0.062	*p* = 0.061	*p* = 0.97
Maternal education^5^								
<5 years	49 (30/61)	80 (51/64)	64 (270/421)	64 (265/416)	39 (163/416)	56 (234/421)	34 (144/421)	36 (151/421)
5-9 years	37 (32/87)	76 (54/71)	65 (321/494)	61 (297/490)	40 (198/490)	57 (282/494)	34 (169/494)	37 (184/494)
≥10 years	16 (10/61)	78 (21/27)	76 (166/220)	54 (118/219)	41 (90/219)	69 (151/220)	42 (92/220)	43 (95/220)
	*p* = 0.001	*p* = 0.88	*p* = 0.009	*p* = 0.06	*p* = 0.88	*p* = 0.004	*p* = 0.107	*p* = 0.18
Household food insecurity^5^								
Highest	41 (28/69)	78 (40/51)	59 (220/372)	63 (229/365)	37 (134/365)	55 (203/372)	30 (110/372)	38 (141/372)
Middle	34 (24/71)	73 (45/62)	68 (259/383)	63 (238/381)	42 (160/381)	57 (217/383)	37 (140/383)	36 (138/383)
Lowest	28 (20/71)	84 (41/49)	73 (281/385)	56 (216/383)	42 (160/383)	65 (249/385)	41 (156/385)	39 (151/385)
	*p* = 0.30	*p* = 0.37	*p* = <0.001	*p* = 0.13	*p* = 0.25	*p* = 0.011	*p* = 0.006	*p* = 0.66

WHO, World Health Organization; ^1^Values are (%) percentages and (n/N); ^2^Criteria based on consumption of at least one food item of four of the seven food groups during the past day; ^3^Based on WHO guidelines considering age and breastfeeding status; ^4^Based on children who had at least one unmet WHO complementary feeding practice and who also consumed at least one highly processed snack or at least one sugar-sweetened beverage; ^5^n/N-values may be larger in housing quality and household food insecurity analyses; Pearson’s chi-squared test was utilized to find differences of feeding practices, children´s consumption of highly processed snacks and sugar-sweetened beverages and children´s exposure to a double burden of suboptimal feeding by the socio-economic resources; Significance level at <0.05.

**Table 3:** **Associations of socio-economic resources and WHO breastfeeding indicators amongst children in Nicaragua**

	**Not met exclusive breastfeeding 0–5 m**			**Not met continued breastfeeding 12–15 m**		
	**Unadjusted**	**Adjusted 1** ^**1**^	**Adjusted 2** ^**2**^	**Unadjusted**	**Adjusted 1** ^**1**^	**Adjusted 2** ^**2**^
**Socio-economic resources**	**OR (95% CI)**	**OR (95% CI)**	**OR (95% CI)**	**OR (95% CI)**	**OR (95% CI)**	**OR (95% CI)**
Housing quality^3^						
Lowest	0.51 (0.24,1.08)	0.57 (0.24,1.36)	1.08 (0.40,2.90)	0.86 (0.32, 2.30)	0.63 (0.21,1.91)	0.71 (0.22, 2.33)
Middle	0.53 (0.25,1.14)	0.60 (0.25,1.43)	0.75 (0.30,1.90)	1.28 (0.50, 3.24)	1.16 (0.43,3.13)	1.08 (0.36, 3.18)
Highest	Ref.	Ref.	Ref.	Ref.	Ref.	Ref.
Maternal education^3^						
<5 years	0.20** (0.09,0.47)	0.20** (0.08,0.51)	0.19** (0.07,0.51)	0.89 (0.30, 2.66)	0.61 (0.19,2.02)	0.65 (0.17,2.47)
5-9 years	0.34** (0.15,0.75)	0.51 (0.21,1.26)	0.50 (0.20,1.26)	1.10 (0.38, 3.18)	1.22 (0.40,3.70)	1.23 (0.36,4.19)
≥10 years	Ref.	Ref.	Ref.	Ref.	Ref.	Ref.
Household food insecurity^3^						
Highest	0.57 (0.28,1.16)	0.54 (0.24,1.21)	0.58 (0.25,1.35)	1.41 (0.51,3.87)	1.22 (0.42,3.54)	1.50 (0.49,4.64)
Middle	0.77 (0.38,1.57)	0.67 (0.30,1.53)	0.71 (0.29,1.69)	1.94 (0.76,4.96)	2.26 (0.83,6.14)	2.37 (0.85,6.59)
Lowest	Ref.	Ref.	Ref.	Ref.	Ref.	Ref.

WHO, World Health Organization; OR, odds ratio; CI, confidence intervals; ^1^Adjusted for child age, maternal age, municipality; ^2^Adjusted for housing quality, maternal education, household food insecurity, child age, maternal age, municipality; ^3^n/N-values may be larger in housing quality and household food insecurity analyses; **Significance level at <0.05.

Maternal education and maternal employment were associated in the study area. When focusing on mothers of the youngest infants (aged 0–5 months), none (0/65) of those with less than 5 years of schooling had employment. This finding can be compared with the 32% (22/68) of mothers with 10 years or more of education (*p* = <0.001). Analyses were also undertaken to evaluate differences in infant feeding between employed and unemployed mothers. None of the employed mothers (0/14) practised exclusive breastfeeding (infants 0–5 m), as compared to 36% of unemployed mothers (70/193) (*p* = 0.006). Further, focusing on the age group of children when employed mothers are covered by maternity leave (infants 0–2 m), the majority (5/7) of employed mothers provided formula to their infants, whilst only 8% (6/76) of unemployed mothers did so (*p* = <0.001).

No significant associations were observed between continued breastfeeding (aged 12–15 months) and the socio-economic resources, either in bivariate or multivariate analyses (Tables 2 and 3).

#### Dietary diversity and meal frequency in children aged 6–35 months

The proportion of children who met the minimum acceptable dietary diversity criteria varied with maternal education (*p* = 0.009), household food insecurity (*p* = <0.001) and housing quality (*p* = 0.012) in bivariate analyses (Table 2). For all three resources a lower level of “beneficial” exposure appeared to be associated with a smaller proportion of children with adequate dietary diversity. After controlling for potential confounders, as well as for the other socio-economic resources, the odds ratios for poor dietary diversity were higher in the lowest and middle educational categories, as well as in households with worst food insecurity (Table 4). The results suggest that maternal education and household food insecurity were mediating resources in the association between housing quality and dietary diversity.

**Table 4:** **Associations of socio-economic resources and WHO complementary feeding indicators amongst children in Nicaragua**

	**Not met minimum dietary diversity** ^**1**^ **6-35 m**			**Not met minimum meal frequency** ^**2**^ **6-35 m**			**Not met minimum acceptable diet 6–35 m**		
	**Unadjusted**	**Adjusted 1** ^**3**^	**Adjusted 2** ^**4**^	**Unadjusted**	**Adjusted 1** ^**3**^	**Adjusted 2** ^**4**^	**Unadjusted**	**Adjusted 1** ^**3**^	**Adjusted 2** ^**4**^
**Socio-economic resources**	**OR (95% CI)**	**OR (95% CI)**	**OR (95% CI)**	**OR (95% CI)**	**OR (95% CI)**	**OR (95% CI)**	**OR (95% CI)**	**OR (95% CI)**	**OR (95% CI)**
Housing quality^5^									
Lowest	1.54** (1.11,2.14)	1.50** (1.05,2.15)	1.20 (0.82,1.74)	0.89 (0.65,1.22)	0.93 (0.67,1.30)	1.11 (0.78,1.58)	1.30 (0.95,1.77)	1.28 (0.92,1.77)	1.24 (0.88,1.75)
Middle	1.10 (0.79,1.55)	1.19 (0.83,1.69)	1.03 (0.72,1.49)	0.89 (0.65,1.22)	0.89 (0.64,1.23)	0.99 (0.70,1.38)	1.10 (0.81,1.50)	1.14 (0.83,1.57)	1.13 (0.82,1.57)
Highest	Ref.	Ref.	Ref.	Ref.	Ref.	Ref.	Ref.	Ref.	Ref.
Maternal education^5^									
<5 years	1.72** (1.19,2.48)	2.31** (1.57,3.42)	2.04** (1.36,3.08)	0.67** (0.48,0.93)	0.60** (0.42,0.85)	0.60** (0.41,0.86)	1.08 (0.78,1.51)	1.20 (0.85,1.69)	1.10 (0.77,1.59)
5-9 years	1.66** (1.16,2.37)	1.81** (1.24,2.64)	1.70** (1.15,2.50)	0.76 (0.55,1.05)	0.75 (0.54,1.05)	0.76 (0.54,1.07)	1.02 (0.74,1.42)	1.07 (0.77,1.49)	1.02 (0.72,1.43)
≥10 years	Ref.	Ref.	Ref.	Ref.	Ref.	Ref.	Ref.	Ref.	Ref.
Household food insecurity^5^									
Highest	1.87** (1.38,2.53)	1.68** (1.21,2.33)	1.47** (1.05,2.05)	0.77 (0.57,1.03)	0.84 (0.62,1.14)	0.89 (0.65,1.21)	1.24 (0.92,1.66)	1.18 (0.87,1.60)	1.13 (0.83,1.54)
Middle	1.29 (0.95,1.77)	1.31 (0.94,1.82)	1.21 (0.87,1.69)	0.78 (0.58,1.04)	0.76 (0.56,1.02)	0.78 (0.58,1.06)	0.99 (0.74,1.32)	0.98 (0.73,1.32)	0.95 (0.71,1.28)
Lowest	Ref.	Ref.	Ref.	Ref.	Ref.	Ref.	Ref.	Ref.	Ref.

WHO, World Health Organization; OR, odds ratio; CI, confidence intervals; ^1^Criteria based on consumption of at least one food item of four of the seven food groups during the past day; ^2^Based on WHO guidelines considering age and breastfeeding status; ^3^Adjusted for child age, maternal age, municipality; ^4^Adjusted for housing quality, maternal education, household food insecurity, child age, maternal age, municipality; ^5^n/N-values may be larger in housing quality and household food insecurity analyses; **Significance level at <0.05.

In bivariate analyses, children’s meal frequency neither varied with maternal education, household food insecurity nor with housing quality (Table 2). When adjusting for confounders and for the other socio-economic resources the odds ratio for poor meal frequency was lower in the lowest maternal education category (Table 4).

#### HP snacks and SSBs and a double burden of suboptimal feeding in children aged 6–35 months

There was a significant association between consumption of HP snacks and maternal education, such that children with mothers in the lowest level of education ate fewer HP snacks (*p* = 0.004) (Table 2). There were also a lower proportion of children in the highest household food insecurity group who consumed HP snacks (*p* = 0.011) and SSBs (*p* = 0.006). In the adjusted analyses, children of mothers with the lowest education were significantly less likely to consume HP snacks and SSBs, as compared to children with mothers of higher education (Table 5). No associations were found between HP snacks and SSBs and housing quality or household food insecurity after adjusting for the other socio-economic resources, indicating that maternal education was a closer determinant of consumption of HP snacks and SSBs than housing quality or household food insecurity.

**Table 5:** **Associations of socio-economic resources and not recommended feeding practices amongst children in Nicaragua**

	**Highly processed snacks 6–35 m**			**Sugar-sweetened beverages 6–35 m**			**Exposed to double burden of suboptimal feeding** ^**1**^ **6–35 m**		
	**Unadjusted**	**Adjusted 1** ^**2**^	**Adjusted 2** ^**3**^	**Unadjusted**	**Adjusted 1** ^**2**^	**Adjusted 2** ^**3**^	**Unadjusted**	**Adjusted 1** ^**2**^	**Adjusted 2** ^**3**^
**Socio-economic resources**	**OR (95% CI)**	**OR (95% CI)**	**OR (95% CI)**	**OR (95% CI)**	**OR (95% CI)**	**OR (95% CI)**	**OR (95% CI)**	**OR (95% CI)**	**OR (95% CI)**
Housing quality^4^									
Lowest	0.75 (0.55,1.02)	0.69** (0.50,0.97)	0.88 (0.62,1.25)	0.68** (0.50, 0.94)	0.67** (0.47,0.94)	0.76 (0.53,1.09)	1.03 (0.75,1.41)	1.00 (0.72,1.39)	1.15 (0.81,1.63)
Middle	1.01 (0.73,1.38)	0.89 (0.64,1.24)	1.04 (0.74,1.46)	0.81 (0.59, 1.11)	0.73 (0.52,1.01)	0.78 (0.56,1.10)	1.04 (0.76,1.43)	1.00 (0.72,1.38)	1.08 (0.77,1.51)
Highest	Ref.	Ref.	Ref.	Ref.	Ref.	Ref.	Ref.	Ref.	Ref.
Maternal education^4^									
<5 years	0.57** (0.41,0.81)	0.43** (0.30,0.61)	0.47** (0.32,0.68)	0.72 (0.52, 1.01)	0.59** (0.41,0.84)	0.68** (0.46,0.98)	0.74 (0.53,1.03)	0.66 (0.46,0.93)**	0.64 (0.44,0.92)**
5-9 years	0.61** (0.43,0.85)	0.53** (0.37,0.75)	0.55** (0.39,0.79)	0.72 (0.52,1.00)	0.65** (0.46,0.91)	0.71 (0.50,1.00)	0.78 (0.57,1.08)	0.75 (0.54,1.04)	0.73 (0.52,1.03)
≥10 years	Ref.	Ref.	Ref.	Ref.	Ref.	Ref.	Ref.	Ref.	Ref.
Household food insecurity^4^									
Highest	0.66** (0.49,0.88)	0.68** (0.50,0.92)	0.78 (0.57,1.08)	0.62** (0.46,0.83)	0.70** (0.51,0.97)	0.76 (0.55,1.05)	0.95 (0.71,1.27)	0.93 (0.68,1.26)	0.98 (0.72,1.33)
Middle	0.71** (0.53,0.96)	0.73** (0.54,0.99)	0.78 (0.58,1.07)	0.85 (0.63,1.13)	0.86 (0.64,1.17)	0.92 (0.68,1.25)	0.87 (0.65,1.17)	0.91 (0.67,1.23)	0.93 (0.69,1.26)
Lowest	Ref.	Ref.	Ref.	Ref.	Ref.	Ref.	Ref.	Ref.	Ref.

OR, odds ratio; CI, confidence intervals; ^1^Based on children who had at least one unmet complementary feeding practice (minimum dietary diversity, minimum meal frequency or minimum acceptable diet) and who also consumed at least one highly processed snack or at least one sugar-sweetened beverage; ^2^Adjusted for child age, maternal age, municipality; ^3^Adjusted for housing quality, maternal education, household food insecurity, child age, maternal age, municipality; ^4^n/N-values may be larger in housing quality and household food insecurity analyses; **Significance level at <0.05.

About one-third of children aged 6–35 months were exposed to a double burden of suboptimal feeding (Table 2). No significant associations were observed between a double burden of suboptimal feeding and the socio-economic resources in the unadjusted models (Table 5). However in both adjusted models, the odds ratio for double burden of suboptimal feeding was lowest in children of mothers with lowest education, when compared to children of highest educated mothers (Table 5).

## Discussion

Of the socio-economic resources studied, maternal education explained more of the variation in both the WHO recommended feeding practices and the potentially non-favourable feeding practices of consuming HP snacks and SSBs. Higher maternal education was associated with lower frequency of exclusive breastfeeding, better dietary diversity and fewer children with appropriate meal frequency. A higher educational level was further associated with more frequent consumption of HP snacks and SSBs and higher odds for exposure to a double burden of suboptimal feeding. Being food insecure was associated with poor dietary diversity whilst housing quality was not independently associated with any of the feeding behaviours.

Exclusive breastfeeding was uncommon in *Los Cuatro Santos,* especially amongst mothers with the highest level of education. This finding is in agreement with results reported from a number of low- and middle-income countries, e.g. from peri-urban Peru and urban Kenya [27,28]. Mothers with lower levels of education may be more likely to exclusively breastfeed because they have the opportunity to breastfeed as they more seldom work outside the home. This finding is in contrast to higher educated mothers who more frequently work away from home, which may have compromised their ability to exclusively breastfeed. For mothers in employment, non-existing or insufficient duration of maternity leave may hinder them from practising exclusive breastfeeding. Further, a negative incentive of breastfeeding may be that in Nicaragua, employees who are registered with the National Social Security Institute, receive free formula for the first six months of their baby’s life [29]. This applies to both fathers and mothers regardless of the duration of maternity leave. Our results show that a majority of employed mothers gave formula to their young infants even during the two-months postpartum period covered by maternity leave. Few of the mothers without employment provided formula to their infants at this young age. Whilst Nicaragua has passed Law 295, which protects and promotes breastfeeding [30], the government is promoting the use of formula by providing free formula and thus violating the International Code of Marketing for Breast-milk Substitutes. Suboptimal breastfeeding may lead to gastro-intestinal and other infections, particularly in a setting such as this where there is limited access to safe drinking water. This would subsequently increase the risk of infants being underweight and stunted [31].

In accordance with findings elsewhere [10,14], infants and children of better educated mothers more frequently had an adequate dietary diversity. Apart from the obvious advantage of maternal education to gain a higher level of general knowledge, which may include knowledge on the importance of a diverse diet for children, it has been suggested that mothers with higher education might be more empowered and have more say in the control of household economic resources [32]. This could lead to greater ability to access food resources including a larger diversity of food items. A higher dietary diversity has, in several studies, been shown to be associated with greater height in children [33,34], and the children may thus have a reduced risk of stunting.

Unfortunately, a higher educational level does not necessarily translate into overall better nutritional knowledge and practice [35]. The associations we observed between higher maternal education and more consumption of HP snacks and SSBs may be due to a larger economic capacity of mothers with higher education to get access to these food items coupled with a lack of knowledge on the inappropriateness of their consumption in young children. Consumption of HP snacks and SSBs are known risk factors for children to be overweight [9,36].

Food insecurity also proved to be independently associated with dietary diversity, where lower household food insecurity was associated with better dietary diversity for the children. This is maybe not surprising as the indicator of food insecurity is built on a set of questions assessing the household’s experience of limitations in access to food in general as well as diversity of food items. Therefore, this association may possibly be interpreted as a limited capacity of food insecure households to buffer their children from poor dietary diversity.

Our housing quality indicator intended to capture the wealth of the household. However, it did not show any independent associations with either recommended infant feeding practices or consumption of HP snacks and SSBs. This may be a bit surprising as some of these feeding behaviours are likely to be associated with purchasing capacity. A potential explanation of this finding may be that housing quality, although reflecting past investments in household assets and resources, may not be a sufficiently good indicator of the present economic resources of the household and thus may not capture the variations needed to detect these associations. However, because housing quality was associated with dietary diversity, HP snacks and SSBs in bivariate analyses, as well as in analyses adjusted for confounding factors, another explanation for the lack of independent associations may be that education and/or food security are more proximal determinants of these feeding practices.

Our results point to patterns of infant feeding in the community that we have coined a “double burden of suboptimal feeding” due to its analogy with the concept of “double burden of malnutrition”. Double burden of suboptimal feeding may result from limitations in practising recommended feeding behaviours, potentially leading to different forms of undernutrition in combination with practices of inappropriate feeding behaviours that may be conducive to children being overweight. The double burden of suboptimal feeding may also occur to a varying degree at different levels; in a community (for example in different socio-economic strata), in a household (in different members) or in an individual (concurrently or over time), where our study has provided evidence of concurrent occurrence in individual children. Different patterns of suboptimal feeding practices appeared amongst the children in relation to the mother’s educational level providing evidence for social stratification of suboptimal feeding practices. Whilst double burden of suboptimal feeding occurred in children to mothers of all educational levels, the odds ratio was highest in the highest level of educational category.

Given the cross-sectional design of the study, an obvious limitation is that we cannot ascertain whether the observed associations between the socio-economic resources and infant and young child feeding are causal. Another, but potentially less important, limitation is that the exclusive breastfeeding prevalence may, despite its low prevalence, have been overestimated as it was based on a 24-hour recall of feeding. Similarly, in anticipation of future development assistance the severity of household food insecurity may also have been overestimated. Further, type of foods was not specified in the question aimed at capturing meal frequency. Thus some mothers could have regarded consumption of HP snacks as eating episodes and counted them in the recall of meal frequency, whilst others may have not. Unless these potential biases were associated with the studied socio-economic resources they would not confound our observed associations. Whilst we found no evidence of this, it cannot be ruled out. A strength of the study is the high coverage of the intended study population. In all, despite these limitations, our results should be considered to be valid and generalizable to other rural contexts experiencing similar socio-economic development and degree of nutrition transition.

## Conclusions

Of the three socio-economic resources studied, maternal education explained most of the variation in both favourable and non-favourable feeding practices. Higher education was associated with both more and less of the favourable feeding practices as well as more of the unfavourable feeding practices, including that of a double burden of suboptimal feeding. Higher educational level is thus no guarantee for better feeding practices. Whilst the pathways for the associations between education and the favourable and non-favourable feeding practices remains to be further elucidated, it appears that the knowledge part may be limited in terms of knowledge of appropriate feeding of infants and young children. Further, it appears that the educational level reflects other contextual factors of importance for infant feeding rather than suggesting a causal relation. Regardless of educational strata, it seems that the children in the community are exposed to both suboptimal practices of the recommended feeding practices as well as exposure to HP snacks and SSBs; that is to say, a dual burden of suboptimal feeding. Consequently, children in these communities are also at risk of a double burden of malnutrition.

## Abbreviations

APRODESE: Asociación para el Desarrollo Económico y Social de El Espino
FFQ: Food frequency questionnaire
HDSS: Health and demographic surveillance system
HP snacks: Highly processed snacks
NGO: Non-governmental organization
SSBs: Sugar-sweetened beverages
Sida: Swedish International Development Cooperation Agency
WHO: World Health Organization
